# Prevalence and antimicrobial susceptibility patterns of *Salmonella* isolates in association with hygienic status from butcher shops in Gondar town, Ethiopia

**DOI:** 10.1186/s13756-015-0062-7

**Published:** 2015-05-15

**Authors:** Legesse Garedew, Zenabu Hagos, Zelalem Addis, Reta Tesfaye, Bidir Zegeye

**Affiliations:** Department of Microbiology, St Paul’s Hospital Millennium Medical College, Addis Ababa, Ethiopia; Faculty of Veterinary Medicine, University of Gondar, Gondar, Ethiopia; School of Biomedical and laboratory Sciences, University of Gondar, Gondar, Ethiopia

**Keywords:** Antimicrobial susceptibility, Butcher shops, Hygiene, *Salmonella*, Gondar

## Abstract

**Background:**

*Salmonella* has been recognized as a major cause of food borne illness associated with meat products worldwide. The wide spread of antimicrobial-resistant *Salmonella* has been a serious global human and animal health problem. The aims of this study were to estimate the prevalence and antimicrobial susceptibility pattern of S*almonella* isolates from butcher shops of Gondar town, Ethiopia.

**Methods:**

A cross-sectional study was conducted from February to June, 2013 in Gondar town. After receiving written consent from the study participants, raw meat and swab samples from butcher shops’ utensils and meat handlers were collected and tested using standard bacteriological methods. The isolates were identified using colony characteristics, Gram-reaction, biochemical reaction and sugar tests. Antimicrobial susceptibility test was performed using Kirby-Bauer disc diffusion method. Check list was used to record general hygienic conditions and practices in the butcher shops. The data was entered and analyzed using SPSS version 20.0.

**Results:**

Butcher shop premises and utensils sanitation and personnel’s hygiene were poor. The overall prevalence of *Salmonella* was 17.3 %. *Salmonella* was detected in 32 (35.6 %) meat samples, 13 (23.2 %) hand swabs, 5 (9.1 %) knife swabs, and 3 (5.6 %) chopping board surface swabs. Of the total 53 *Salmonella* isolates subjected to antimicrobial susceptibility test, 47 (88.7 %), 35 (62.3 %), 19 (35.8 %), 17 (32.1 %) and 16 (30.2 %) of them exhibited resistance to Ampicillin, Amoxicillin, Nitrofuranthoin, Tetracycline, and Sulfamethoxazole-Trimethoprime, respectively. Furthermore; 15 (28.3 %) of the isolates were multidrug-resistant from which highest isolation was recorded from meat samples and 40 (75.5 %) isolates of *Salmonella* showed resistance to two or more antimicrobial agents tested. Interestingly, all of the isolates were susceptible to Gentamycin and Ceftriaxone.

**Conclusion:**

The general sanitary condition of the butcher shops and utensils used and personnel hygiene were not to the recommended standards. Contamination of meat, knives, and meat handlers hand with *Salmonella* was found high. Furthermore; multidrug-resistant *Salmonella* is circulating in the butchers’ shop which is strong enough to warrant the revision of butcher shops sanitation policy and treatment regimen for infections implicated by *Salmonella* in the study area. Further in-depth study including serotyping and antimicrobial resistant gene identification is recommended.

## Background

Food borne diseases are public health problems both in developed and developing countries. Thousands of millions of people fall ill and may die as a result of eating unsafe food [1]. Biological contaminants largely bacteria, constitute the major cause of food borne diseases [2]. *Salmonella* is one of the leading causes of human gastroenteritis. There exist many factors that contribute to this development. Among these are the adaptive ability of the pathogen itself, the changing characteristics of the population, the increasing globalization of the food trade, and changes in industrial structure and in consumer behavior [3].

The meat from affected animals may contain *Salmonella* and so cause food contamination in humans [4]. *Salmonella* also inhabit the intestinal tract of a wide range of the common meat animal species. Physical contamination of the carcass or organs by stomach or intestinal contents is a significant route of transmission for *Salmonella* [4, 5]. Contamination with *Salmonella* can occur during production, transportation, preparation, storage and service [6]. At butchers’ house, meat contamination could occur due to different possible reasons; storing food in unclean utensils, holding food at a temperature that would permit microbial growth, utilization of water of questionable hygienic quality, using packaging materials that is not of food-grade quality, vending site that had no facilities for waste disposal and utilization of unclean utensils. In addition, lack of awareness in basic personal cleanliness and safe food handling of butchers enhances contamination of meat by microbes [7]. Consumers should be able to assume that all food offered for sale is safe for its intended use. Primary responsibility for food safety lies with those who produce, process and trade including farmers, slaughterhouse operators, food processors, wholesale and retail traders, caterers, etc [8].

Generally, the increased application of antimicrobials in veterinary and human medicine has been implicated as a contributing factor in the emergence of antimicrobial-resistant pathogens and the evolution of multiple drug-resistant strains. The increased level of drug resistant *Salmonella* has become a problem in all countries, though the extent varies. Developing countries tend to have a high level of resistant *Salmonella* [3, 9, 10].

Retailing raw meat at butcher shops is widely being practiced in different towns of Ethiopia including Gondar town. Bucher shops are the main supplier of meat of different animals such as cattle, sheep and goat to the public. To the authors’ best knowledge; there is no report on the microbial quality, hygienic status and practices of butcher shops in the current study area despite the population’s routine habit of consuming raw meat. It is therefore, considered pertinent to study the prevalence of *Salmonella* and its susceptibility patterns to different antimicrobial agents commonly used in human medicine. Besides, it is also important to assess the hygienic status and practices in the butcher shops.

## Materials and methods

### Study area

This study was conducted in Gondar town. Gondar is located northwest of Ethiopia at latitude and longitude of 12°36’N and 37°28’E. Its altitude is 2200 m above sea level. The maximum and minimum temperatures of the area are 30.7 °C and 12.3 °C, respectively. The area receives a bimodal rainfall pattern with the annual precipitation rate being 1000 mm. The total human population of the town is estimated to be 206,987. According to the information obtained from Gondar town trade & industry office there are about 90 butcher shops in the town [11].

### Study design and population

A cross-sectional study was conducted from February to June, 2013 to estimate the prevalence of *Salmonella* isolates and their susceptibility to antimicrobial agents and to assess hygienic status and practices in butcher shops at Gondar town. The study populations were all Butcher shops operating in Gondar town during the study period.

### Sample size

A total of 306 samples were collected from the 90 butcher shops. Ninety samples were raw meat from meat displayed for vending using census sampling method. The remaining 216 swab samples were from noses and hands of meat handlers, and knives and chopping boards of the butcher shops.

### Ethical considerations

The study protocol was reviewed and approved by Institutional Review Board of School of Biomedical and Laboratory Sciences, College of Medicine and Health Sciences, University of Gondar. Written informed consents were obtained from each participant after informing about the purpose of the study, the procedure, the risk, benefit and their right. All the information obtained from the study participants was kept confidential.

### Data collection and laboratory analysis

#### Observational check list

Observational check list was developed after reviewing relevant literatures to assess the butcher shops’ hygienic status and practice. The check list incorporated food handling practices of workers and hygienic conditions of the butcher shops’ premises and utensils.

### Sample collection and transportation

Fresh raw meat samples of cattle origin were collected from different portion of the carcass, following most purchasers’ preference to keep uniformity, in separate sterile plastic bags. All of meat samples were cattle meat since there was no shoat meat sold during our study period which is uncommon in general in Gondar town. Swab samples were taken from 15-20 cm^2^ of the surface of meat-cutting equipment such as knives and wooden boards. Swab samples were also collected from hands and noses of meat handlers. The collected swab samples were put into sterile test tube. The samples were then transported to University of Gondar, Faculty of Veterinary Medicine, Veterinary Microbiology Laboratory, using a cold box and processed immediately.

### Laboratory methods

#### Enrichment

Twenty five grams of raw meat was homogenized in 225 ml of 0.1 % buffered peptone water by shaking for five minutes in a sterile stomacher. The homogenate was then incubated at a temperature of 37 °C for 24 h for enrichment. Then, 1 ml aliquot of homogenate was transferred to sterile test tube containing 9 ml distilled water to make appropriate serial dilutions for the microbiological analysis. The swab samples were incubated in 5 ml brain heart infusion broth at a temperature of 37 °C for 24 h for enrichment [12, 13].

### Isolation and identification of *Salmonella* isolates

For the isolation of *Salmonella*, 0.1 ml aliquot of homogenate of meat samples from the appropriate dilutions and a loopful of the swab specimen were directly inoculated on to MacConkey agar (Oxoid, England) and Salmonella-Shigella (Oxoid, England) agar incubated aerobically at 37 °C for 24–48 h. Characteristic colonies of *Salmonella* were sub-cultured on brain heart infusion agar and identified using triple sugar iron (TSI), lysine iron agar (LIA), urea, motility, citrate utilization, oxidase tests, indole production, methyl red (MR), Voges-Proscauer (VP), and recommended sugar tests to confirm suspected isolates [12, 13].

### Antimicrobial susceptibility test

*Salmonella* isolates were subjected to *in-vitro* susceptibility test against commonly used antimicrobial agents using disk diffusion method following guidelines established by the Clinical and Laboratory Standards Institute (CLSI) [14]. In brief, by taking pure isolated colony, bacterial suspension was adjusted to 0.5McFarland turbidity standards. The diluted bacterial suspension was then transferred to Mueller-Hinton agar plate using a sterile cotton swab and the plate was seeded uniformly by rubbing the swab against the entire agar surface followed by 24 h incubation. After the inoculums were dried, antibiotic impregnated disks were applied to the surface of the inoculated plates using sterile forceps. The plates were then incubated aerobically at 37 °C for 24 h. *E. coli* (ATCC 25922), which was susceptible to all tested drugs, was used for quality control. Finally, the zone of inhibition was measured including the disk diameter. The susceptible, intermediate and resistant categories were assigned on the basis of the critical points recommended by the CLSI and according to the manufacturer’s leaflet attached to the disks.

Antimicrobials used for susceptibility testing of *Salmonella* species were Amoxicillin (AML-2 μg), Ampicillin (AMP-10 μg), Tetracycline (TE-30 μg), Gentamicin (CN-10 μg), Sulfamethoxazole-Trimethoprim (SXT-25 μg), Ceftriaxone (CRO-30 μg), Nitrofuranthoin (F-100 μg), and Nalidixic acid (NA-30 μg). All the antibiotic disks were purchased from Oxoid Ltd (Basingstoke, Hampshire, England) and the criteria used to select the antimicrobial agents were antimicrobial disks availability and CLSI protocol [14].

### Data analysis

Data was entered and analyzed using the statistical software SPSS version 20.0. Descriptive statistics was used to describe the frequency of *Salmonella* from different sampling points, antimicrobial susceptibility pattern and hygienic conditions.

## Results

### Hygienic status of the butcher shops and meat handlers

Out of the 90 butcher shops assessed in this research, the floor of 60 (66.7 %) were constructed from earthen materials, 23 (25.6 %) were constructed from concrete and the remaining were made of tile and wooden materials. More than half (54.4 %) of the butcher’s shops floor had cracks. Twenty four (26.7 %) of the butcher shops did not have shelf to display meat. Sixty three (70 %) of the shops were with no refrigerator for meat preservation. Majority (N = 84, 93.3 %) of the butcher shops’ chopping board were made of wooden materials of which 60 (66.7 %) were not smooth and easily washable. Sixty five (72.2 %) of the butcher shops meat hanger and knives were not clean. All of the butcher shops were using plastics, newspapers and/or papers to wrap meat while selling. Among the 90 butcher shop workers, only 6.7 % of them wore clean protective clothing during meat handling. None of the butchers had taken any form of formal training on food safety and hygiene. There was also no any hand wash basin within any of the shops for hand washing to minimize contamination as tabulated in Table 1.

**Table 1:** Hygienic status of the butcher shops premises, utensils and meat handlers (N = 90)

	Checkpoints	Frequency	%
Floor is constructed of:	Concrete	23	25.6
	Tile	3	3.3
	Earthen material	60	66.7
	Wood	4	4.4
Floor free of big cracks		41	45.6
Having ceiling		61	67.8
Wall and ceiling free of dust		33	36.7
Wall painted white color paint		49	54.4
Having shelf for meat display		66	73.3
Insect proof shelf		43	65
Refrigerator available		27	30
Chopping board is made up of:	Wooden material	84	93.3
	Concrete	1	1.1
	Marble	4	4.4
	Plastic	1	1.1
Chopping board washable		30	33.3
Clean meat hanger		25	27.8
Clean knives		25	27.8
Material to wrap meat:	Plastic bag	32	35.6
	Newspaper	33	36.7
	Used paper	24	26.7
Personal hygiene: clean clothing and clean hands		6	6.7
Handling money with bare hands		90	100
Food handling training		0	0
Presence of first aid kits		0	0
Wash hand basin nearby		0	0

### Prevalence of *Salmonella*

In the current study, 90 meat samples of cattle, and each 54 swab samples from hands, knifes, chopping boards and nose of meat handlers were assessed for contamination by *Salmonella* and the overall prevalence was 17.3 %. It was detected in 32 (35.6 %) meat samples, 13 (24.1 %) hand swabs, 5 (9.1 %) knife swabs, and 3 (5.6 %) chopping board surface swabs. No *Salmonella* was recovered from nasal swabs. The higher isolation rate was observed among meat samples than other samples analyzed in this study. All of the *Salmonella* isolates identified from hand swabs in this investigation were from butcher shops workers who did not have hand washing habit after using toilet, after touching dirty materials and before handling meat and equipments.

### Antimicrobial susceptibility patterns of *Salmonella* isolates

Of the total 53 *Salmonella* isolates subjected for antimicrobial susceptibility test, 47 (88.7 %), 35 (62.3 %), 19 (35.8 %), 17 (32.1 %) and 16 (30.2 %) exhibited resistance to Ampicillin, Amoxicillin, Nitrofuranthoin, Tetracycline, and Sulfamethoxazole-Trimethoprime in that order. The worrying aspect of the current study is that 15 (28.3 %) of the isolates were multidrug-resistant out of which 9 (60.0 %), 4 (26.6 %), 1 (6.7 %) and 1 (6.7 %) were from meat, hand, knife and chopping board, respectively. Besides, 40 (75.5 %) isolates of *Salmonella* showed resistance to two or more antimicrobial agents tested. Interestingly, all of the isolates were susceptible to Gentamycin and Ceftriaxone as indicated in Tables 2 and 3.

**Table 2:** Antimicrobial susceptibility patterns of *Salmonella* isolates

Antimicrobials	*Salmonella* species (n = 53)		
	Sensitive (N (%))	Intermediate (N (%))	Resistant (N (%))
AML	15 (28.3)	5 (9.4)	35 (62.3)
AMP	2 (3.8)	4 (7.5)	47 (88.7)
TE	27 (50.9)	9 (17)	17 (32.1)
CN	53 (100)	0 (0)	0 (0)
SXT	37 (69.8)	0 (0)	16 (30.2)
NA	51 (96.2)	0 (0)	2 (3.8)
CRO	50 (94.3)	3 (5.7)	0 (0)
F	18 (34)	16 (30.2)	19 (35.8)

**Table 3:** Drug-resistance profile *Salmonella* isolates

Resistance profile	No of isolates with resistance profile (N (%))	Resistance category
AMP-AML-TE-SXT-F	2 (3.8)	Multidrug resistant
AMP-AML-TE-SXT	2 (3.8)	Multidrug resistant
AMP-AML-SXT-F	2 (3.8)	Drug resistant
AMP-AML-TE-F	3 (5.7)	Multidrug resistant
AMP-TE-SXT-F	2 (3.8)	Multidrug resistant
TE-SXT-NA-F	1 (1.9)	Multidrug resistant
AMP-AML-SXT	3 (5.7)	Drug resistant
AMP-TE-SXT	1 (1.9)	Multidrug resistant
AMP-AML-TE	1 (1.9)	Drug resistant
AMP-AML-F	4 (7.5)	Drug resistant
AMP-TE-F	2 (3.8)	Multidrug resistant
AML-TE-F	2 (3.8)	Multidrug resistant
AMP-AML	9 (17)	Drug resistant
AMP-SXT	2 (3.8)	Drug resistant
AMP-TE	2 (3.8)	Drug resistant
AMP-F	1 (1.9)	Drug resistant
AML-F	1 (1.9)	Drug resistant

Key: AML = Amoxicillin, AMP = Ampicillin, TE = Tetracycline, CN = Gentamycin, SXT = Sulfamethoxazole-Trimethoprime, NA = Nalidixic Acid, CRO = Ceftriaxone, F = Nitrofuranthoin

## Discussion

In the current study, majority of butcher shops floor was not constructed of materials that help cleaning. In addition; more than half of the butcher shops floor had cracks, nearly half of the walls of the shops were not painted with white color and one-third of the shops did not have a ceiling which further hinder cleaning. Moreover, most of the walls and ceilings were dusty. These conditions in the present study settings strongly disagreed with the standard. Structures within food establishments should be soundly built of durable materials and be easy to maintain, clean and where appropriate, able to be disinfected. In this regard, WHO and FAO recommend the following specific conditions to be satisfied where necessary to protect the safety and suitability of food: the surfaces of floors and walls should be made of impervious materials with no toxic effect in intended use; floors should be constructed to allow adequate drainage and cleaning; walls should have a smooth surface up to a height appropriate to the operation; ceilings and overhead fixtures should be constructed and finished to minimize the buildup of dirt and condensation, and the shedding of particles [15].

The result of this study showed that a good number of the butcher shops did not have shelf to display meat. Off those who had shelves, one-third of them were not insect proof. About 70 % of the shops were with no refrigerator for meat preservation. The majority of the butcher shops’ chopping board was made of wooden material with rough surface, and meat hangers and knives were not clean. Surprising enough, none of the butcher shops used an appropriate type of paper to wrap meat. This is again against the requirement that all equipment and areas within food premises must be kept clean, and carcasses should go into the cooler as soon as possible to retard bacterial growth and extend the shelf-life [15, 16].

In this assessment, it was also observed that less than 7 % of the butcher shops workers wore clean protective clothing during meat handling. In almost all shops no any wash hand basin within the shops, personnel handled money with bare hand while serving meat, and no first aid kit. This might be attributed to lack of knowledge about good hygienic practices as none of the butchers had taken formal training in food safety. It is a well documented fact that poor personal hygiene is one of the most important sources of contamination for foods. [15, 16]. Simultaneous handling of food and money increases the risk of cross contamination [17].

The frequency of occurrence of *Salmonella* in the raw meat in this study was 35.6% which was the highest isolation rate compared with hand, knife, chopping board and nose samples in the current study. This is higher than 7 %, 9 % and 14.8 % observed in retail or butcher shops in Karachi, Pakistan [18], Awasa, Ethiopia [19] and India [20] respectively. But it is lower than 42 % report from minced meat in Addis Ababa [21] and 54 % report from raw retailed meat in Yucatan, Mexico [22]. This difference in the prevalence of *Salmonella* could be due to differences in the sanitation of butcher shops premises and hygienic standards of meat handlers.

*Salmonella* prevalence observed in the current study from hand swabs (23.2 %) was by far higher than reports from hands of workers (8.9 %) in eviscerating area in Modjo abattoir, Ethiopia [23]. Personal hygiene differences of the food handlers might help to explain this discrepancy since majority of workers in the current investigation settings practiced very poor hand washing. Improvement of food worker hand washing practices, the least costly intervention to implement, is critical to the reduction of food borne illness [24].

The prevalence (9.1 %) of *Salmonella* observed in the current study in cutting knife was higher than reported from Mojo, Ethiopia [23] and slightly lower than from Botswana abattoir [25]. The current high prevalence recorded in the cutting knife might be due to the high prevalence of *Salmonella* in meat samples which might act as a source of contamination for the knives and due to poor hygienic condition of knives.

Relatively lower prevalence *(*5.6 %*)* of *Salmonella* was registered from chopping board surfaces than in meat samples, knife swabs and hands of the meat handlers in this study. This might be ascribed to the nature of cutting board. Researchers described that bacteria such as *Salmonella* were not recoverable from wooden surfaces in a short time after they were applied, unless very large numbers were used. It was indicated that wood is intrinsically porous, which allows food juices and bacteria to enter the body of the wood unless a highly hydrophobic residue covers the surface [26].

Antimicrobial susceptibility test results of the current study indicated that the highest number (60 %) of multidrug resistant isolates was identified from meat samples followed by hand samples (26.7 %). In addition, the finding of this research revealed that more than one-fourth (28.3 %) of *Salmonella* isolates were multidrug-resistant according to the recent drug-resistance definition [27]. *Salmonella* resistance pattern for Ampicillin was comparable among isolates identified from different samples in the current study and with reports from Iran, India, Nigeria and Cameroon [28–30]. The high resistance observed to antimicrobials including Ampicillin, Amoxicillin, Nitrofuranthoin, Tetracycline, and Trimethoprime-Sulfamethaxazole in this study could be due to uncontrolled availability of the antimicrobial agents in drug vendors, which leads to misuse. Thus, this might exert greater selection pressure for the resistant strains thereby making them resistant to antimicrobials. The presence of antimicrobial resistance have the potential to adversely affect human health by causing illness that is more difficult to treat because of the resistance profile of the microorganism. The current study reported almost similar resistant *Salmonella* isolates identification rate from different samples for each antimicrobial exposed except higher resistance observed among isolates from meat samples to tetracycline. This higher resistance profile of *Salmonella* isolates to tetracycline might be attributed to high level of utilization of this drug both in veterinary and human medicines due to its relatively cheaper price and readily availability to the local community in the current study area. Prevalence of multidrug-resistance *Salmonella* isolates identified in this investigation was higher (28.3 %) even though lower compared to a report from Bahir Dar, Ethiopia [31]. More than half and about quarter of drug resistance isolates were sourced from meat and hand samples, respectively. But there was no extensively drug-resistant and pandrug-resistant isolates recovered currently.

In the present research, all of the *Salmonella* isolates were susceptible to Gentamycin and Ceftriaxone which is consistent with other studies in Iran and Ethiopia [28, 31–33]. Very good (96.2 %) efficacy was observed for Nalidixic acid. In contrary to this study, 75 % to 96 % resistance rates to Nalidixic acid were reported from Modjo and Bahir Dar, Ethiopia [23, 33].

## Conclusion

The higher prevalence of *Salmonella* isolates from raw meat and other critical control points in this study gives a warning signal for possible occurrence of food borne infections capable of producing outbreaks. The poor personnel hygiene and low sanitary standard of butcher shops and their premises observed may have contributed to the high prevalence of *Salmonella*. Besides, moderate to higher degree of drug-resistance and multidrug-resistance was exhibited for antimicrobials which are in frequent use for both human and veterinary medicine in Ethiopia. Therefore, these situations are enough to warrant urgent and strict intervention to protect the community. Principles of hazard analysis critical control points should be applied at butcher shops to prevent contamination. Training should be planned and implemented to improve the knowledge, attitude and practice of the butcher shops personnel about food safety. The problem of drug resistance should also be considered through using drug susceptibility tests before treating patients and efforts should be in place to avoid microbial resistance to antimicrobials. Further in-depth serotyping and drug resistant gene identification should be carried out.
